# Synthesis and characterization of HDPE/N-MWNT nanocomposite films

**DOI:** 10.1186/1556-276X-9-288

**Published:** 2014-06-09

**Authors:** Fairouz Chouit, Ounassa Guellati, Skander Boukhezar, Aicha Harat, Mohamed Guerioune, Nacer Badi

**Affiliations:** 1 Laboratoire d'Etude et de Recherche des Etats Condensés (LEREC), Département de Physique, Université Badji-Mokhtar, BP. 12, Annaba 23000, Algeria; 2 Laboratoire des Matériaux, Surfaces et Procédés pour la Catalyse (LMSPC), ECPM - CNRS, UdS, 25 rue Becquerel, Strasbourg 67087, France; 3 Université Mohamed Chérif Messaadia, BP. 1553, Souk-Ahras 41000, Algeria; 4 Center for Advanced Materials, University of Houston, Houston, TX 77204-5004, USA

**Keywords:** Nanocomposite, Carbon nanotube, High-density polyethylene, Raman spectroscopy, XRD, SEM

## Abstract

In this work, a series of nitrogen-doped multi-walled carbon nanotubes (N-MWCNTs) with several weight percentages (0.1, 0.4, 0.8, and 1.0 wt.%) were synthesized by catalytic chemical vapor deposition (CCVD) technique. The N-MWCNTs were first characterized and then dispersed in high-density polyethylene (HDPE) polymer matrix to form a nanocomposite. The HDPE/N-MWCNT nanocomposite films were prepared by melt mixing and hot pressing; a good dispersion in the matrix and a good N-MWCNT-polymer interfacial adhesion have been verified by scanning electron microscopy (SEM). Raman spectroscopy measurements have been performed on prepared samples to confirm the presence and nature of N-MWNTs in HDPE matrix. The X-ray diffraction (XRD) analysis demonstrated that the crystalline structure of HDPE matrix was not affected by the incorporation of the N-MWNTs.

## Background

The carbon nanotubes (CNTs) have attracted a great attention in recent years in the field of nanocomposite materials as reinforcing fillers because of their excellent mechanical and thermal properties [1,2]. They possess an extremely high elastic modulus comparable to that of diamond [3,4]. In addition, they exhibit electrical conductivity as high as 10^5^ to 10^7^ S/m [5] and can transform an insulating polymer into a conducting composite at a very low loading due to their extremely high aspect ratio. The CNT/polymer nanocomposite is one of the most promising fields for CNT applications, which generally exhibits excellent properties that differ substantially from those of pristine polymer matrix. A good dispersion of CNTs in polymer and their strong interfacial adhesion or coupling are the two key issues to ensure success of fabricating CNT/polymer nanocomposite with excellent properties [6,7]. To that end, CNT functionalization is necessary before compounding with polymers. Three general approaches have been adopted in attempts to modify the surface of CNTs to promote the interfacial interactions: chemical, electrochemical, and plasma treatments. For example, Velasco-Santos et al. [8] placed different organofunctional groups on MWCNTs using an oxidation and silanization process. Bubert et al. [9] modified the surface of CNTs by using low-pressure oxygen plasma treatment. They detected hydroxide, carbonyl, and carboxyl functionality on the surface layers of the CNTs by using X-ray photoelectron spectroscopy (XPS).

Polyethylene (PE) is one of the most widely used thermoplastic. Among all PE types, high-density polyethylene (HDPE) is a commonly used thermoplastic with high degree of crystalline structure along with higher tensile strength [10-12]. Due to its low cost and processing energy consumption, HDPE resin is ideal for many applications such as orthopedic implants and distribution pipes [11]. Moreover, HDPE can effectively resist corrosions including moisture, acids/alkalis, and most of the chemical solvents at room temperature.

High-power ultrasonic mixers [13], surfactants, solution mixing [14], and *in situ* polymerization have been used to produce CNT/polymer composites. These techniques appear to be environmentally contentious and may not be commercially viable. The melt mixing technique reported here is a simple and economical approach since the nanofillers are added directly to the polymer melt. However, the challenge in melt mixing is to achieve a good dispersion of the nanofillers through shear forces as well as a strong coupling between nanofillers and the matrix [15].

It has been shown that CNTs can alter the crystallization kinetics of semi-crystalline polymers [16,17]. Sandler et al. [18] have melt-blended polyamide-12 with MWCNTs and carbon fibers using a twin-screw micro-extruder, and then fibers were produced from the prepared blends. They highlighted that both the intrinsic crystalline quality of the nanocomposite and the orientation of the embedded CNTs are the major factors controlling the reinforcing capability of CNTs.

We report in this paper on the preparation of nitrogen-doped multi-walled carbon nanotube (N-MWNT)/high-density polyethylene (HDPE) composites using melt blending. The presence of N-MWNTs in HDPE and morphology of the composites were investigated using scanning electron microscopy (SEM) and Raman spectroscopy techniques. The crystallization of the nanocomposites is subsequently discussed using X-ray diffraction combined with Raman analysis.

## Methods

### Materials

The main materials used in this study are N-MWNTs (> 97% purity) with an outer mean diameter around 40 nm and a length over 10 μm. These nanotubes were synthesized by catalytic chemical vapor deposition (CCVD) technique using a mixture of C_2_H_6_/Ar/NH_3_ and 20 wt.% iron catalyst supported by alumina powder. The polymer matrix used is HDPE with trade name TR144, supplied by Sonatrach Company CP2K (Skikda, Algeria). The melt index of HDPE pellets is 0.30 with a density of 0.942 to 0.947 g/cm^3^.

### Nanocomposite preparation

N-MWNTs/HDPE were prepared via the melt-compounding method using a twin-screw mixer (Brabender, Duisburg, Germany), the processing temperature was kept at 167°C, and the screw speed amounted to 100 rpm for 10 min. The weight fractions of N-MWNT filler were fixed at 0.1, 0.4, 0.8, and 1.0 wt.%. The composite was then hot-pressed at 177°C, under a pressure of 100 bars for 5 min, in order to obtain films using 50 × 70 × 0.5 mm^3^ mold dimensions. In addition, a reference sample of bare HDPE was prepared in a very similar way.

### Characterization techniques

The morphology of the N-MWNTs was examined by SEM on a JEOL 6700-FEG microscope (Akishima, Tokyo, Japan). High-magnification transmission electron microscopy (HRTEM) observations were carried out using a JEOL JEM-2010 F under an accelerated voltage of 200 kV with a point-to-point resolution of 0.23 nm. The thermogravimetric analysis (TGA) was performed on a Q5000 apparatus (TA Instruments, New Castle, DE, USA) where the combustion ran in air atmosphere at a flow rate of 20 ml*/*min, up to 1,000°C at 10°C*/*min. Raman spectroscopy was carried out on a micro-Raman Renishaw spectrometer Ramascope 2000 (Gloucestershire, UK), with a spot size of 1 μm^2^, a resolution of 1 cm^-1^, and a He-Ne laser beam operating at an excitation wavelength of 632.8 nm. X-ray diffraction measurements have been performed by PANalytical system (Almelo, The Netherlands; Cu_Kα_ as a radiation source with *λ* = 1.0425 Ǻ, 2*θ* from 10° to 60°).

## Results and discussions

### Analysis of carbon nanotubes

**Figure 1:**
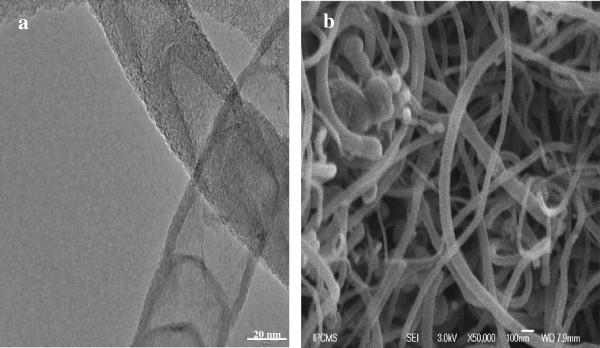
HR-TEM (a) and SEM (b) micrographs of N-MWNTs.

SEM studies give further information on the morphology and microstructure of the prepared N-MWNTs. Figure 1 is a typical magnification HRTEM image of the synthesized product showing the bamboo-shaped MWNTs with 97% purity and high selectivity (approximately 12 to 100 nm) with an outer diameter around 40 nm [19,20].

**Figure 2:**
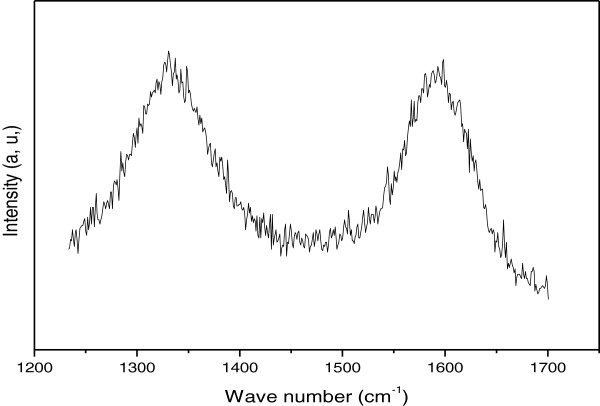
Raman spectrum of N-MWNTs.

**Figure 3:**
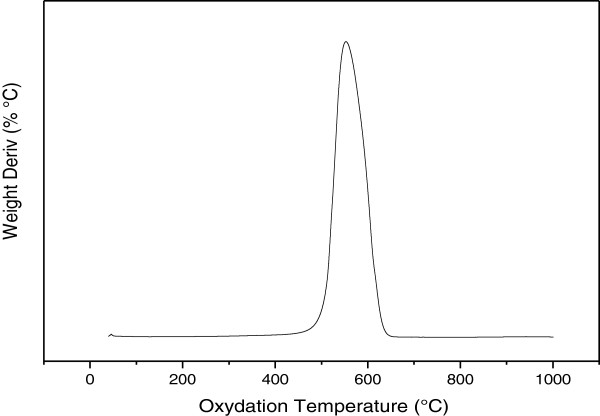
Derivative of TGA curve of N-MWNTs.

In order to obtain more information regarding the nitrogen doping and crystallinity of the entire nitrogen-doped MWNTs, Raman spectrum is plotted in Figure 2. It shows two main features: the D and G bands. The first band at around 1,331 cm^-1^ originated from atomic displacement and disorder caused by structural defect [21]. The second one at around 1,599 cm^-1^ indicates the graphitic state of bamboo MWNTs. Moreover, the intensity ratio of D to G (*I*_D_/*I*_G_) is measured to be 1.14. This suggests a certain degree of orderly graphitic structure in the prepared nitrogen-doped MWNTs, which is consistent with the observed TEM results.The TGA is used to investigate the distribution and species of the carbon phases present in CNTs. Figure 3 shows the derivative of TGA curve of the nitrogen-doped MWNTs. The weight loss is considered due to the combustion of carbon in air atmosphere and represents more than 97% of carbon content for the prepared sample with oxidation peak at 550°C. Consequently, this shift in the mass loss maxima suggests more defects and disorders for the nitrogen-doped MWNTs which are in good agreement with the Raman results.

### Characterization of nanocomposites (HDPE/N-MWNTs)

**Figure 4:**
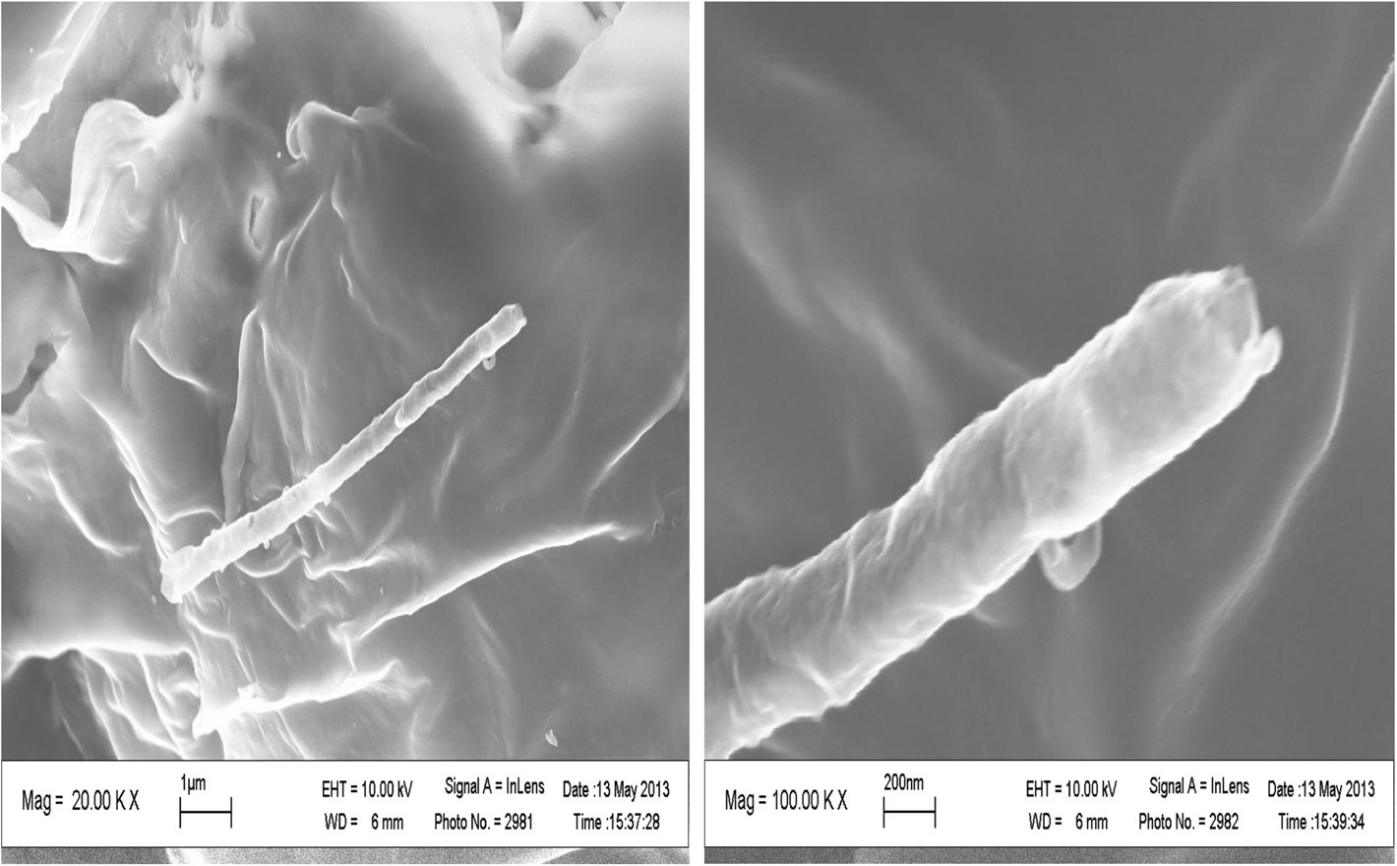
SEM micrographs of HDPE/N-MWNT nanocomposite.

**Figure 5:**
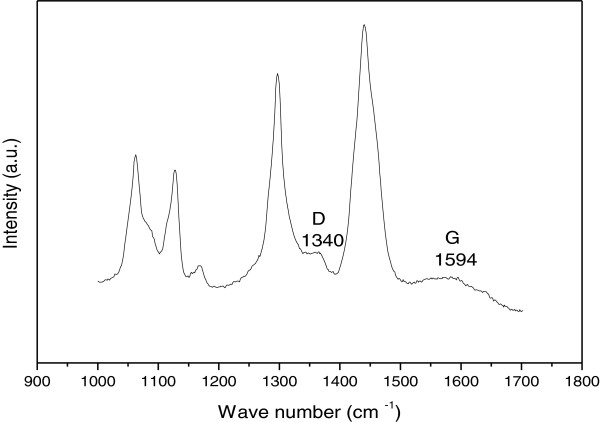
Raman shift of HDPE/N-MWNT nanocomposite.

The SEM images for the nanocomposites were taken without any treatment at two different magnifications. The nanocomposite cross-sectional surface for 0.8 wt.% N-MWCNT content is represented in Figure 4, where the N-MWNT in HDPE is clearly observed even at low loadings of MWNT in the composites.The Raman analysis for this nanocomposite presented in Figure 5 shows the presence of the D and G bands in the background as a result of the relatively low concentration of MWNT in polymer. However, the presence of carbon nanostructures can still be easily detected, and their Raman feature peaks are located at similar bandwidth as the ones in the pristine material.

On the other hand, the larger intensity reflections are the bands resulting from the HDPE matrix as reported in the literature [22]. The band at 1,080 cm^-1^ is used to characterize the level of amorphous phase in HDPE. Indeed, Raman spectroscopy is one of the most powerful tools to characterize the crystallinity of HDPE [22], and this is made through the intensity measurement between 1,400 and 1,460 cm^-1^. Those bands are characteristics of the methylene bending vibrations. In particular, the band in the 1,418 cm^-1^ region is typically assigned to that of the orthorhombic crystalline phase in polyethylene [22-24].

**Figure 6:**
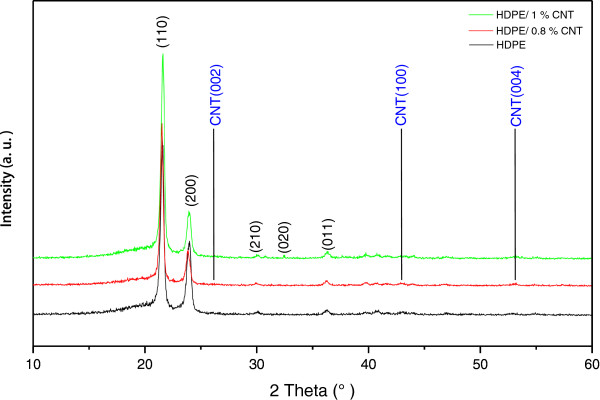
X-ray patterns of HDPE and HDPE/N-MWNTs.

Furthermore, Figure 6 shows the X-ray diffraction (XRD) patterns of the pristine HDPE and nanocomposites filled with N-MWNTs. The pristine HDPE mainly exhibits a strong reflection peak at 21.6° followed by a less intensive peak at 24.0°, which correspond to the typical orthorhombic unit cell structure of (110) and (200) reflection planes, respectively. These 2*θ* values are in good agreement with the reported values of polyethylene [10,25,26]. The two weak peaks at 2*θ* around 30.0° and 36.2° are attributed to reflection planes (210) and (020), respectively [27,28]. In addition, there are several other weak reflection planes in the range of 38° to 60° [28]. The two crystalline characteristic peaks (110) and (200) remain unchanged after the incorporation of the N-MWNTs, indicating that the addition of the N-MWNTs did not affect the original crystal structure of the HDPE matrix.

## Conclusion

A melt processing method has been used to prepare HDPE/N-MWNT nanocomposites with different filler loading percentages between 0.1, 0.4, 0.8, and 1.0 wt.%. The CNTs were dispersed into the host HDPE matrix by shearing action only of a pair of cylinder screws and then hot-pressed. HRTEM observations indicate that the N-MWNT product exhibits a bamboo shape with 97% purity and a high selectivity. The presence of N-MWNT in polymer matrix HDPE is clearly observed even at low loadings of N-MWNTs. The fraction of the crystalline phase was determined from the normalized integrated intensity of the 1,418 cm^-1^ Raman band, which represents the orthorhombic crystalline phase in polyethylene. The XRD analysis demonstrated that the crystalline structure of HDPE matrix was not affected by the incorporation of the N-MWNTs.

## Competing interests

The authors declare that they have no competing interests.

## Authors’ contributions

MG conceived the idea and planned the experiments. FC carried out the synthesis of nanocomposites and their characterization and analyzed the data. OG carried out the synthesis of carbon nanotubes and their characterization. NB carried out the Raman spectroscopy and analyzed the data. The manuscript was prepared by FC. NB, OG, MG, and SB, and AH helped with the draft editing and contributed to the preparation and revision of the paper. All authors read and approved the final manuscript.
